# Characterization of a Maize *Wip1* Promoter in Transgenic Plants

**DOI:** 10.3390/ijms141223872

**Published:** 2013-12-06

**Authors:** Shengxue Zhang, Yun Lian, Yan Liu, Xiaoqing Wang, Yunjun Liu, Guoying Wang

**Affiliations:** 1 Institute of Crop Sciences, Chinese Academy of Agricultural Sciences, Zhongguancun South Street 12, Beijing 100081, China; E-Mails: xueshzhang@163.com (S.Z.); lyer09@163.com (Y.L.); xiaoqingshiyan@126.com (X.W.); 2 Institute of Industrial Crops, Henan Academy of Agricultural Sciences, Zhengzhou 450002, Henan, China; E-Mail: lianyun262@126.com

**Keywords:** *Wip1*, promoter, transcriptional start site, transgenic plant

## Abstract

The Maize *Wip1* gene encodes a wound-induced Bowman-Birk inhibitor (BBI) protein which is a type of serine protease inhibitor, and its expression is induced by wounding or infection, conferring resistance against pathogens and pests. In this study, the maize *Wip1* promoter was isolated and its function was analyzed. Different truncated *Wip1* promoters were fused upstream of the *GUS* reporter gene and transformed into *Arabidopsis*, tobacco and rice plants. We found that (1) several truncated maize *Wip1* promoters led to strong GUS activities in both transgenic *Arabidopsis* and tobacco leaves, whereas low GUS activity was detected in transgenic rice leaves; (2) the *Wip1* promoter was not wound-induced in transgenic tobacco leaves, but was induced by wounding in transgenic rice leaves; (3) the truncated *Wip1* promoter had different activity in different organs of transgenic tobacco plants; (4) the transgenic plant leaves containing different truncated *Wip1* promoters had low *GUS* transcripts, even though high GUS protein level and GUS activities were observed; (5) there was one transcription start site of *Wip1* gene in maize and two transcription start sites of *GUS* in *Wip1::GUS* transgenic lines; (6) the adjacent 35S promoter which is present in the transformation vectors enhanced the activity of the truncated *Wip1* promoters in transgenic tobacco leaves, but did not influence the disability of truncated *Wip*_1231_ promoter to respond to wounding signals. We speculate that an ACAAAA hexamer, several CAA trimers and several elements similar to ACAATTAC octamer in the 5′-untranslated region might contribute to the strong GUS activity in *Wip*_1231_ transgenic lines, meanwhile, compared to the 5′-untranslated region from *Wip*_1231_ transgenic lines, the additional upstream open reading frames (uORFs) in the 5′-untranslated region from *Wip*_1737_ transgenic lines might contribute to the lower level of *GUS* transcript and GUS activity.

## 1. Introduction

It is necessary to use different promoters when multiple genes are cloned in tandem in transgenic plants because multiple copies of a single promoter could lead to the silencing of the transgenes. Promoters may be classified as constitutive, tissue-specific, or inducible based on their expression patterns. The most widely used constitutive promoters include the CaMV 35S promoter [1], the maize ubiquitin promoter [2], and the rice actin promoter [3]. Several other strong promoters have also been isolated and identified from plants or viruses [4–6]. Although many promoters have been isolated and tested, only a few have been successfully used in agricultural biotechnology. The constitutive expression of certain genes might be harmful to their host plants, affecting plant growth and development; inducible or tissue-specific promoters would be more useful than constitutive promoters in these cases.

The function of a promoter is normally determined by the combinatorial action of multiple regulatory elements, *i.e.*, enhancers and *cis*-elements, and by the interactions between regulatory elements and nuclear protein factors [7]. For the CaMV 35S promoter, the region from −46 to +1 upstream of the gene is the basal promoter and the region from −343 to −46 acts as an enhancer [1]. Although the CaMV 35S promoter is presumed to be constitutive, some reports have shown that it is developmentally regulated [8] and can be affected by abiotic stress [9]. Negative *cis*-regulatory regions determine the tissue-specific activity of a number of promoters [10]. Besides the *cis* elements in DNA sequence affect transcription, various *cis* elements in mRNA have the functions to control the translation efficiency. Many reports have shown that the 5′-untranslated region (5′-UTR) and 3′-untranslated region (3′-UTR) can increase the translation efficiency [11]. Upstream open reading frames (uORFs) that are located in the 5′-UTR have been reported to generally downregulate the expression of the major open reading frame (mORF) through ribosomal stalling or reducing initiation efficiency [12]. However, a few uORFs can upregulate the expression of mORF [13]. Additionally, more and more reports showed that the 35S promoter interferes with the tissue specificity, strength [14] or inducibility (our data, unpublished) of its adjacent promoter. The onset and extent of influence depends on the responsive nature of the adjacent promoter and the distance between the 35S promoter and adjacent promoter [15].

The Bowman-Birk inhibitor (BBI) is a type of serine protease inhibitor, and its expression is induced by wounding or infection, conferring resistance against pathogens and pests [16]. It was reported that the BBI confers heavy metal and multiple drug tolerance in yeast [17]. There are seven BBI genes in rice and overexpression of the rice BBI2-3 conferred fungal pathogen resistance in transgenic rice plants [18]. The maize *Wip1* gene, encoding a wound-induced BBI protein, has been cloned, and the promoter of the gene might respond to wound signals [19,20]. In contrast to most BBIs, which inhibit trypsin and chymotrypsin proteases, both of the inhibitory domains of maize *Wip1* inhibit chymotrypsin [20]; however, these two domains have different evolutionary histories and ecological functions [21].

Here, we isolated the maize *Wip1* promoter and characterized its function in transgenic plants, showing that *Wip1* promoters showed different properties in dicot and monocot plants, and some truncated promoters under the influence of adjacent 35S promoter can drive high GUS activities even with a low *GUS* transcriptional level.

## 2. Results

### 2.1. Isolation and Analysis of the Maize *Wip1* Promoter

**Figure 1.**
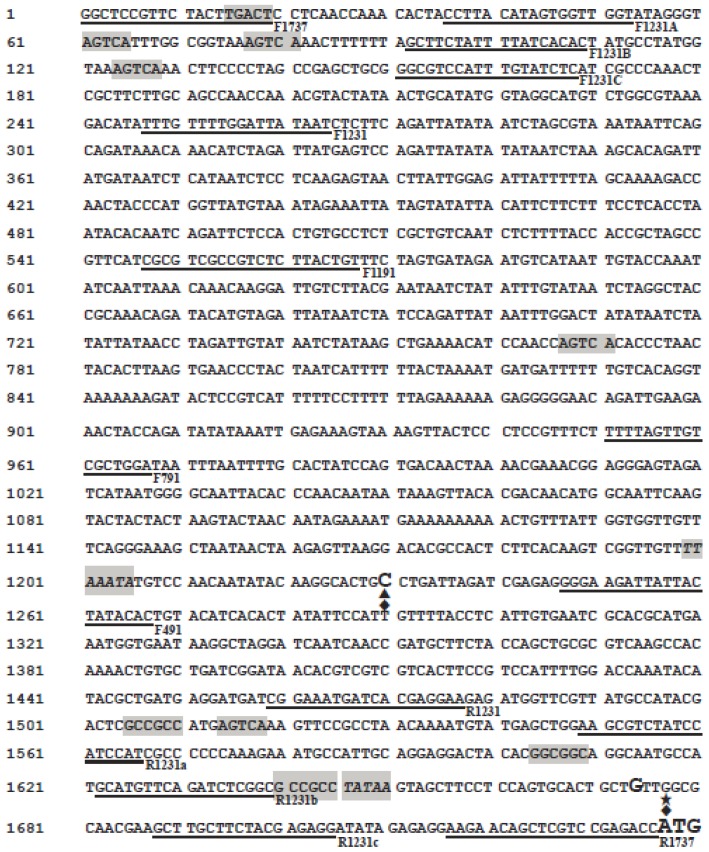
The maize *Wip1* promoter sequence. The start codon (ATG) and putative transcription start sites are indicated in larger letters. The putative TATA boxes are shadowed and italicized. The W-box and GC-box are shadowed only. The primers used for PCR amplification are underlined and given corresponding names. Triangle (▲) indicates the transcription start site of *GUS* in *Wip*_1231_*::GUS* line; rhombuses (♦) indicate the transcription start site of *GUS* in *Wip*_1737_*::GUS* line; asterisk (★) means the transcription start site of *Wip1* in maize.

To isolate the maize *Wip1* promoter, we performed a BLAST analysis with the mRNA sequence of maize *Wip1* (X71396) in www.maizegdb.org [22]. The maize *Wip1* sequence was homologous to the genome sequence from 12,274,189 to 12,273,525 on chromosome 8, and one intron was found in the *Wip1* coding region. Based on the maize genomic sequence, a 1737 bp fragment (chr8: 12,275,876–12,274,140) upstream of the translation start site of *Wip1* was amplified by PCR. The *cis*-elements in the promoter sequence were analyzed using the PlantCARE [23] and PLACE [24] databases, and the transcription start sites were predicated on the website: http://www.fruitfly.org/seq_tools/promoter.html [25]. Two putative TATA boxes and two corresponding potential transcription start sites were present in the promoter sequence. Six W-boxes and three GCC-boxes were found in the *Wip1* promoter. In addition, several CAAT-boxes, which are common *cis*-acting elements in promoter and enhancer regions, were also found (Figure 1).

### 2.2. Truncated *Wip1* Promoter Has Strong Activity in Transgenic *Arabidopsis* and Tobacco Leaves

**Figure 2.**
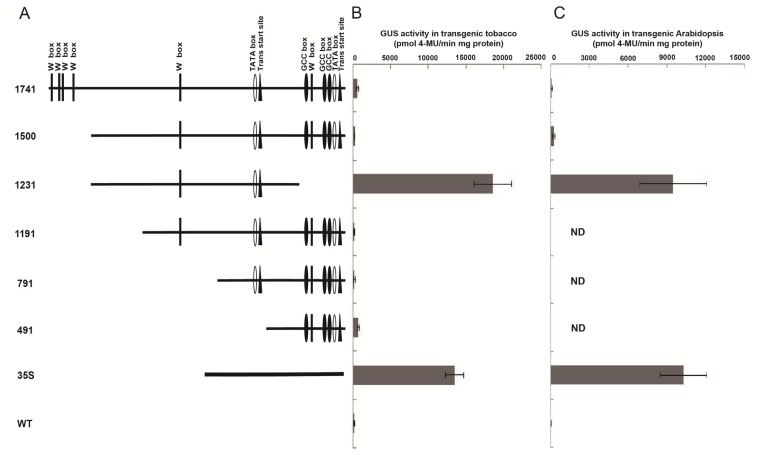
The *Wip1* promoters and their activities in transgenic tobacco and *Arabidopsis* plant leaves. (**A**) Schematics of the full-length and truncated *Wip1* promoters. The numbers and/or letters in front of each schematic represent the names of the corresponding constructs; (**B**) The GUS activities of 40-day-old transgenic tobacco plants containing different constructs. Error bars represent the S.E. of *n* independent transgenic lines, *n* = 9 for 1737 and 1500; *n* = 16 for 1231 and 35S; *n* = 3 for 1191, 791, 491 and WT; and (**C**) The GUS activity of 20-day-old transgenic *Arabidopsis* plants. Error bars represent the S.E. of *n* independent transgenic lines, *n* = 6 for 1737 and 1500; *n* = 20 for 1231 and 35S; *n* = 3 for WT. ND indicates not determined.

**Figure 3.**
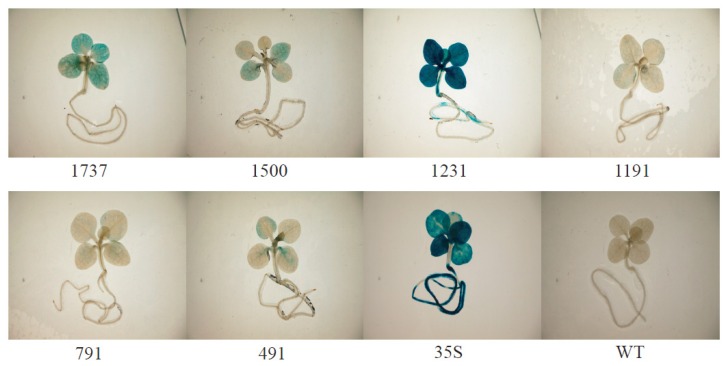
Histochemical GUS staining of 14-day-old transgenic tobacco plants. T_2_ transgenic tobacco seeds were germinated on MS medium plates with 30 mg/L Hygromycin B to eliminate the non-transgenic plants. The surviving 14-day-old seedlings were immersed in GUS staining solution and incubated overnight at 37 °C. Then, the samples were de-pigmented with 70% ethanol at 37 °C until the chlorophyll had completely disappeared.

To analyze the function of the *Wip1* promoter, several expression vectors (pWip_1737_, pWip_1500_, pWip_1231_, pWip_1191_, pWip_791_, pWip_491_) were constructed (Figure 2A, Tables 1 and 2). The constructed plasmids were transformed into tobacco, and at least ten independent transgenic tobacco lines for each construct were obtained. The transformations were confirmed by PCR analysis of genomic DNA using primers specific for *GUS* (data not shown). We performed quantitative analysis of GUS activity using leaf samples from 40-day-old T_3_ progeny of plants. The results showed that the *Wip*_1231_ promoter had strong activity, while no or weak activity was detected for the other truncated promoters (Figure 2B). To confirm the results of the GUS activity analysis, the seedlings were subjected to histochemical staining. Strong GUS staining was observed for the seedlings that were transgenic for the *Wip*_1231_ promoter and 35S promoter, whereas no or light staining was observed for the seedlings that were transgenic for the full-length and other truncated promoters (Figure 3), consistent with the GUS activity analysis results. To investigate whether the truncated *Wip*_1231_ promoter also had strong activity in other plant species, the constructs were transformed into *Arabidopsis*. As expected, the *Wip*_1231_ promoter also drove high GUS activity in transgenic *Arabidopsis* plant leaves (Figure 2C), further confirming the high activity of the truncated *Wip*_1231_ promoter.

**Figure 4.**
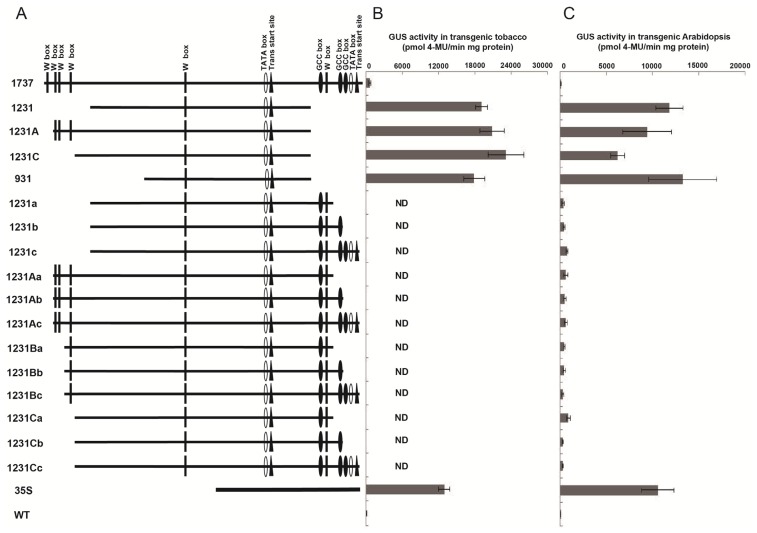
The finely truncated *Wip1* promoters and their activity in transgenic *Arabidopsis* and tobacco plant leaves. (**A**) Schematics of the finely truncated *Wip1* promoters. The numbers or/and letters in front of each schematic indicate the names of the corresponding constructs; (**B**) The GUS activities of transgenic tobacco plants. Error bars represent the S.E of *n* independent transgenic lines, *n* = 8 for 1737, 1231 and 35S; *n* = 18 for 1231A, 1231C and 931; *n* = 3 for WT. ND indicates not determined; and (**C**) The GUS activities of transgenic *Arabidopsis* plants containing different constructs. Error bars represent the S.E. of *n* independent transgenic lines, *n* = 3 for WT, and *n* = 6 for the others.

To find out the potential negative *cis*-acting elements, detailed 5′ and 3′ deletions of *Wip*_1737_, based on *Wip*_1231_, were performed, involving the generation of an additional 15 deletion constructs (pWip_1231A_, pWip_1231C_, pWip_931_, pWip_1231a_, pWip_1231b_, pWip_1231c_, pWip_1231Aa_, pWip_1231Ab_, pWip_1231Ac_, pWip_1231Ba_, pWip_1231Bb_, pWip_1231Bc_, pWip_1231Ca_, pWip_1231Cb_, pWip_1231Cc_; Figure 4A, Tables 1 and 2). These constructs were transformed into *Arabidopsis*, and the GUS activity in the transgenic plant leaves was analyzed. The results showed that *Wip*_1231A_, *Wip*_1231C_, and *Wip*_931_, which were of the same 3′ end as *Wip*_1231_, had high activity similar to that of *Wip*_1231_, while the other *Wip1* promoter fragments presented very low levels of activity (Figure 4C). To further confirm the functions of these three fragments, three constructs (pWip_1231A_, pWip_1231C_ and pWip_931_) were also transformed into tobacco, respectively. High GUS activities were observed in the transgenic tobacco leaves transformed with each of these three promoters (Figure 4B).

### 2.3. *Wip1* Promoters Are not Induced by Wounding and Have Different Activity in Different Organs in Transgenic Tobacco

**Figure 5.**
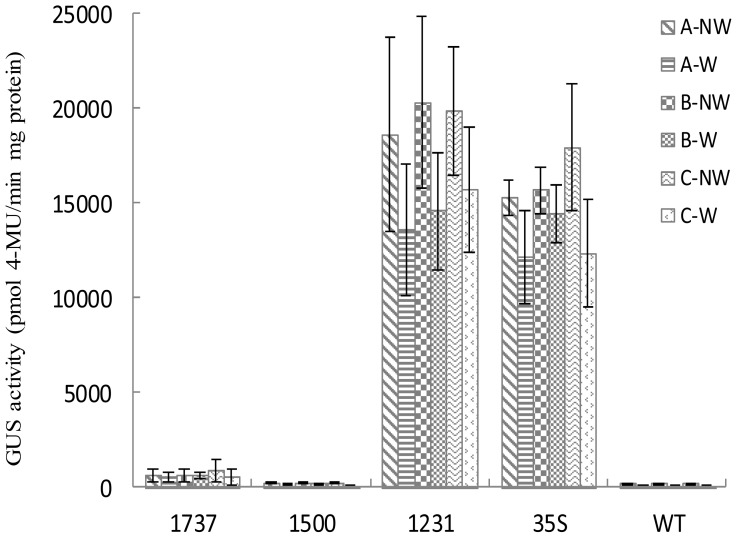
GUS activity in transgenic tobacco leaves treated with wounding. The second leaves from apex of 40-day-old T_3_ tobacco plants grown in greenhouse were wounded using three different kinds of methods. GUS activities were assessed 24 h after wounding. A-NW and A-W mean non-wounded or wounded samples using the method described in Walker-Simmons, *et al.* [26]. B-NW and B-W mean non-wounded or wounded samples using the method described in An, *et al.* [27]. C-NW and C-W mean non-wounded or wounded samples using the method described in Xu, *et al.* [28]. Data was shown as average ± S.E. of 6 independent transgenic lines.

It has been shown that the expression of *Wip1* gene is wound-induced in maize [19,20]. We investigated whether the *Wip1* promoter is wound-induced in transgenic tobacco plant leaves that were wounded with three different kinds of wounding methods, respectively. Under three different treatment conditions, to our surprise, wounding did not induce the increase of the activity of *Wip1* promoters (Figure 5), indicating that *Wip1* promoters are not wound-induced in transgenic tobacco.

**Figure 6.**
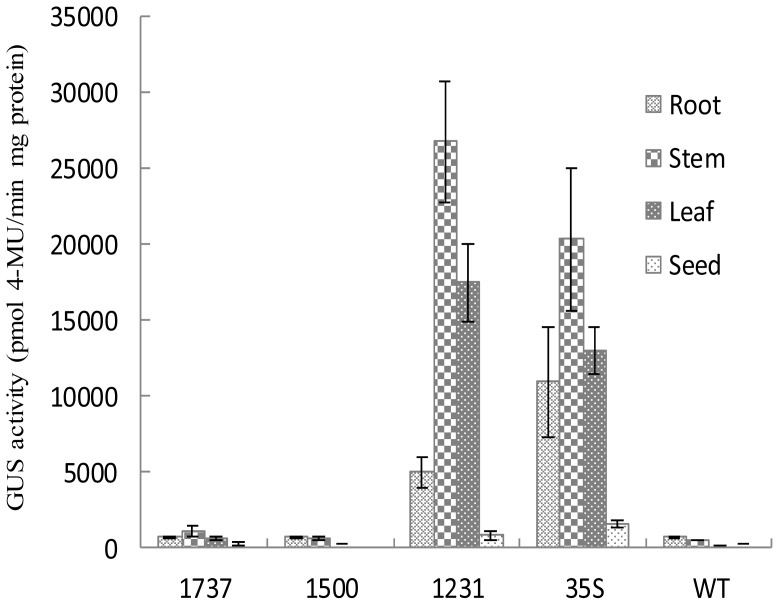
GUS activity of different tissues of transgenic tobacco plants. Data was shown as average ± S.E. of *n* independent transgenic lines, *n* = 3 for 1737, 1500 and WT; *n* = 6 for 1231 and 35S.

The GUS activities in different organs of the transgenic tobacco lines transformed with different truncated *Wip1* promoters or the control 35S promoter were analyzed. The results showed that the *Wip*_1231_ promoter has high activity in the leaf and stem but low activity in the root and seed (Figure 6).

### 2.4. *Wip*_1231_ Promoter Drives Low GUS Transcriptional Level

**Figure 7.**
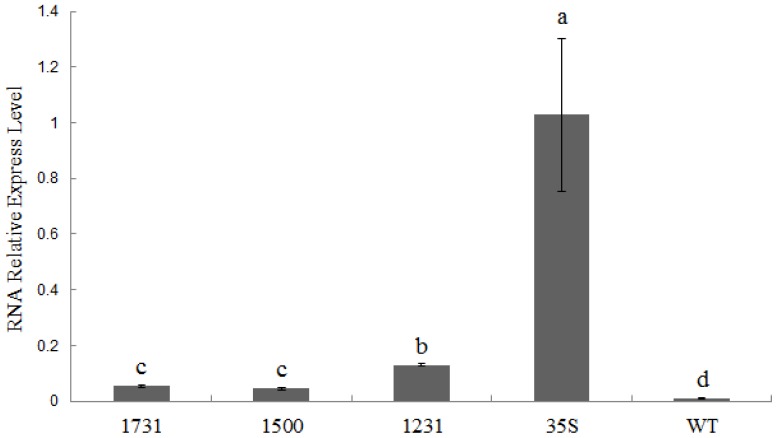
Transcriptional level of *GUS* in transgenic tobacco leaves. Data was shown as average ± S.E. of 6 independent transgenic lines. Letters above the columns indicate statistically significant differences at *p* < 0.05 level. Relative transcript levels were calculated using the 2^−ΔΔ^*^Ct^* method [29] with *Actin* as a housekeeping gene.

Generally, the level of protein is consistent with the level of mRNA, but sometimes they are discordant. To confirm *Wip*_1231_ is a strong promoter, we investigated the transcriptional levels of *GUS* in the transgenic tobacco plants. To our surprise, lower transcriptional levels of *GUS* were detected in transgenic plants containing *Wip1* promoters than in transgenic plants containing CaMV 35S promoter. There were higher *GUS* transcriptional level in *Wip*_1231_*::GUS* transgenic lines than in *Wip*_1737_*::GUS* and *Wip*_1500_*::GUS* lines (Figure 7). The results of Western blot analysis showed that there were high GUS protein levels in both *Wip*_1231_*::GUS* and *35S::GUS* transgenic lines, whereas GUS protein was undetectable in *Wip*_1737_*::GUS*, *Wip*_1500_*::GUS* and WT plants (Figure S1). These results indicate that the strong GUS activity in *Wip*_1231_*::GUS* transgenic lines can be attributed to high efficient translation of GUS protein. We speculate that there might be some translation enhancer in the 5′-UTR of *GUS* transcripts in *Wip*_1231_*::GUS* transgenic plants.

### 2.5. *Wip1* Promoters Have Multiple GUS Transcription Start Sites in Transgenic Tobacco

**Figure 8.**
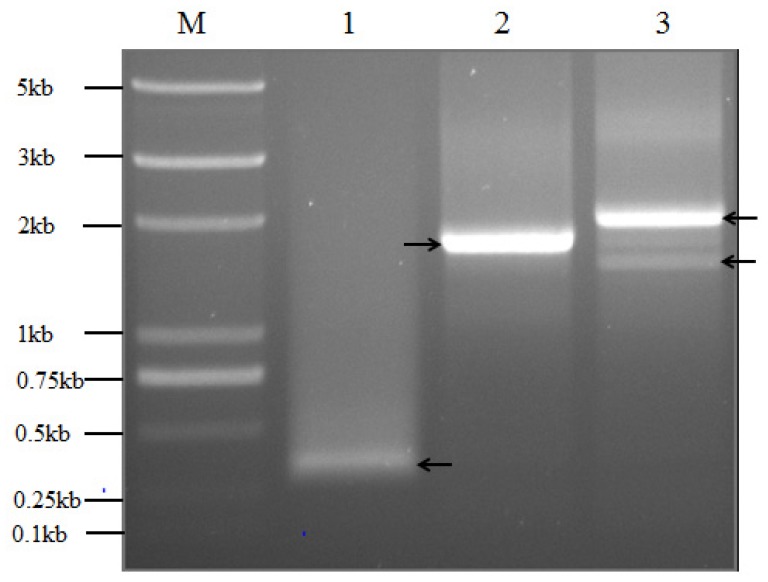
5′-RACE analysis of the transcription start sites. **M**, DNA molecular marker; (**1**) *Wip1* in maize; (**2**) *Wip*_1231_*::GUS* line; and (**3**) *Wip*_1737_*::GUS* line.

To find out the exact 5′-UTRs, we further determined the accurate transcription start sites in maize and transgenic tobacco plants by the 5′-RACE method. The native transcription start site of *Wip1* in maize was identified as the nucleotide G which is located at 61 bp upstream of the *Wip1* translation initiation site and 3 bp downstream of the predicated one (Figures 1 and 8). For *Wip*_1737_*::GUS* transgenic tobacco line, a strong band and one weak band were amplified, and sequencing results revealed that two transcription start sites are present (Figures 1 and 8). The latter is the same as the transcription start site of *Wip1* gene in maize. The former was identified as nucleotide C which is located at 532 bp upstream of the ATG translation initiation site and identical to the predicated one, and this transcription start site was also identified in *Wip*_1231_*::GUS* transgenic tobacco line. So there is a 272 bp-length untranslated region in *GUS* transcripts in *Wip*_1231_*::GUS* lines. In the 272 bp 5′-UTR there is an ACAAAA element, several CAA trimers and several elements similar to ACAATTAC octamer (Figure S2). The elements might contribute to the high translation efficiency of *GUS* transcripts in *Wip*_1231_ transgenic lines. The sequences of 5′-UTR from *Wip*_1231_ and *Wip*_1737_ transgenic lines were comparatively analyzed to find that there were more uORFs in the latter (Figures S3–S5). Maybe the additional uORFs lead to the lower level of *GUS* transcript and GUS activity in *Wip*_1737_ transgenic lines.

### 2.6. *Wip*_1231_ Promoters Were Influenced by the Adjacent 35S Promoter Sequence

**Figure 9.**
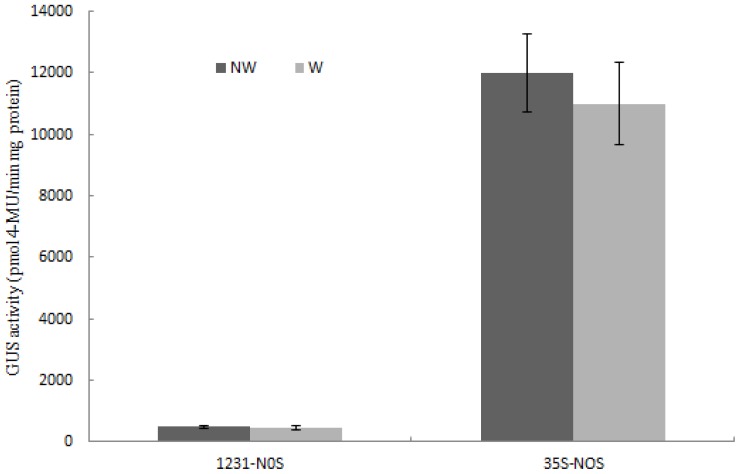
GUS activity in tobacco plant leaves transformed with constructs p1300-35S-NOS or p1300-1231-NOS. The second leaves from apex of 40-day-old T_3_ tobacco plants grown in greenhouse were used. NW and W mean non-wounded or wounded samples using the method described in Walker-Simmons, *et al.* [26]. Data was shown as average ± S.E of *n* independent transgenic lines, *n* = 15 for p1300-1231-NOS; *n* = 13 for p1300-35S-NOS.

In this study, the transformation vectors were constructed from a derivative of the pCAMBIA1300 binary vector that includes a CaMV 35S promoter controlling a *hptII* selectable marker gene. It has been reported that the CaMV 35S enhancer can influence the expression of a transgene within the same transformation construct [14,30,31]. To investigate whether the 35S enhancer influences the truncated *Wip*_1231_, we constructed transformation vectors p1300-1231-NOS and p1300-35S-NOS, where the 35S promoter was replaced with a NOS promoter to control the *hptII* selectable marker gene, and the reporter gene *GUS* was controlled by the *Wip*_1231_ promoter and 35S promoter, respectively. Then they were transformed into tobacco, and transgenic tobacco plants were obtained. The GUS activity in transgenic plants transformed with p1300-1231-NOS was very low, compared to transgenic lines transformed with pWip_1231_. However, transgenic plants transformed with p1300-35S-NOS still had high GUS activity, which were similar to the transgenic lines transformed with p1300-221 (Figure 9). The *Wip*_1231_ promoter was still not induced by wounding in transgenic tobacco plants which was transformed with p1300-1231-NOS (Figure 9). The above results showed that adjacent 35S promoter did enhance the expression of *GUS* reporter gene that was controlled by truncated *Wip*_1231_, and the truncated *Wip*_1231_ does not respond to wound signals in transgenic tobacco plants.

### 2.7. *Wip1* Promoter Is Wound Inducible in Transgenic Rice Plants

**Figure 10.**
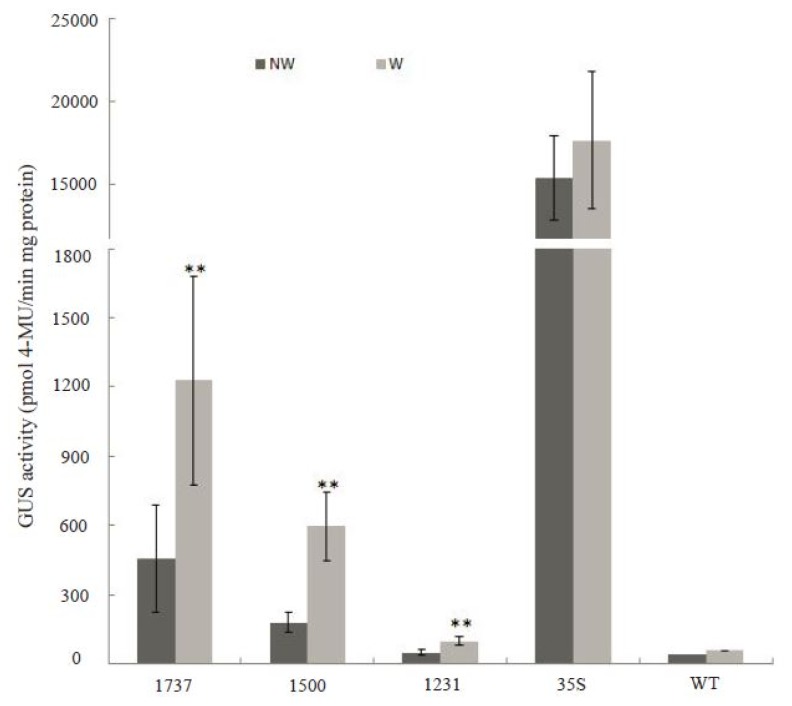
GUS activity in transgenic rice leaves treated with wounding. The second leaves from apex of 40-day-old rice plants grown in a greenhouse were wounded using the method described in Xu, *et al.* [28]. NW and W mean non-wounded or wounded samples. Data was shown as average ± S.E. of 6 independent transgenic lines. Asterisks (**) means the significant difference at *p* < 0.01 level.

Expression of the *Wip1* gene is induced through wounding in maize. However, the *GUS* gene, driven by *Wip1* promoters, was not induced by wounding in transgenic tobacco which is a dicot. This encouraged us to analyze whether *Wip1* promoters have the same function in monocot species. Constructs pWip_1737_, pWip_1500_ and pWip_1231_ were transformed into rice, respectively, and the transgenic rice events were confirmed by PCR analysis of the genomic DNA (data not shown). Low GUS activities were detected in transgenic rice plant leaves containing different *Wip1* promoters, compared with that in transgenic rice transformed with the CaMV 35S promoter. When transgenic rice leaves were wounded, significant increase of GUS activity were observed in all the transgenic lines containing different *Wip1* promoters, whereas the GUS activity in transgenic plants containing the 35S promoter did not change (Figure 10). This indicates that the *Wip1* promoter is induced by wounding in rice.

## 3. Discussion

*Arabidopsis* and tobacco have long been used as simple and high-efficiency transformation systems in which to analyze gene function. In addition to protein-coding genes, the promoters from rice or maize have been studied in one of these two systems [32–34]. In this study, we used both systems to study the function of maize *Wip1* promoter. To our surprise, expression of *GUS* driven by truncated maize *Wip1* fragments was not induced by wound in transgenic tobacco plants (Figure 5). This may be due to the fact that the maize *Wip1* promoter comes from a monocot, and its function may be different in monocot and dicot species. Although it has been reported that the wound-inducible *PinII* promoter from potato was able to drive gene expression in response to wound signals in rice [28], many plant promoters exhibit different functionality in dicots and monocots. For example, the widely used maize ubiquitin promoter conferred lower expression of a reporter gene in the tobacco (dicot) protoplasts than in maize (monocot) protoplasts [2]. Several dicot promoters also have been shown to have lower activities than monocot promoters when transformed into monocot species [35].

Few strong promoters have been identified to date, and it remains important for researchers in plant biotechnology to identify promoters that are as strong as or stronger than the CaMV 35S promoter. In transgenic plants, high GUS activities were observed in transgenic lines containing truncated *Wip1* promoters even though low *GUS* transcriptional levels were detected. The above results indicate that truncated *Wip1* promoters are not strong, and the reason for the high GUS activity driven by the truncated *Wip1* promoters might be due to the increased translation of the *GUS* gene. It has been shown that the *35S-GUS* and *35S-ABI4-GUS* lines had at least 50 fold differences in transcript level, however more than 300 fold difference in GUS activity was observed [36], indicating that transcriptional level is not correlated with the protein level in some cases. Many factors, *i.e.*, promoter activity, mRNA stability, protein translation efficiency, can affect the expression level of foreign genes in transgenic plants. The high GUS activity driven by truncated *Wip1* promoters indicate that *Wip1* promoters are also useful in plant biotechnology, even though these promoters led to low transcriptional level.

Normally, a promoter shows different properties in monocot and dicot plants. It has been shown that the CaMV 35S promoter can drive high levels of transgene expression in dicot plants, whereas its activity is relatively lower in monocot plants [37]. In transgenic rice, lower activity of the *Wip*_1231_ promoter compared to the 35S promoter was observed, and wound treatment significantly increased GUS activities in transgenic rice containing different *Wip1* promoters. These results are contrary to the results in transgenic tobacco, indicating that the *Wip1* promoter also showed different properties in monocot and dicot plants; this will limit the usage of the Wip1 promoter as a general (cross-species) promoter in transgenic crops.

5′-RACE results revealed that only one native transcription start site was present in the maize *Wip1* gene, and a new one was identified in both *Wip*_1737_*::GUS* and *Wip*_1231_*::GUS* transgenic tobacco lines. We analyzed the 272 bp 5′-UTR of *GUS* mRNA from *Wip*_1231_*::GUS* transgenic tobacco lines to find that there is an ACAAAA hexamer, several separate CAA trimers and several elements similar to ACAATTAC octamer (Figure S2). The ACAAAA hexamer exists in many 5′-UTRs of plant genes and has been speculated to enhance translation [38]. The poly(CAA) region and ACAATTAC octamer have been confirmed to enhance translation [39–41]. The features of the sequence may contribute to the property of the 5′-UTR for high efficient translation. It has been reported that uORF plays an important role in the regulation of gene expression by different mechanisms, for example, by ribosomal stalling, reducing initiation efficiency [12]. uORF can downregulate gene expression by accelerating mRNA degradation or upregulating gene expression by reforming the 5′-UTR through the product of the uORF [42]. Six upstream ATGs (uATGs) were found in the 272 bp UTR. Among the six uATGs, the first three are in two very short uORFs, the fourth and fifth are in the reading frame of the *GUS* gene and will produce GUS fusion protein, and the sixth uATG should not have the opportunity to initiate translation (Figure S3). We suggest that the two uORFs may have little possibility to influence the mORF translation. Strong GUS activity in transgenic plants transformed with pWip_1231_ construct and related pWip_1231A_, pWip_1231C_ and pWip_931_ might be due to that they all have the same 3′ end and produce the same 5′-UTR containing two uATGs which produce GUS fusion proteins, and strong translation may initiate from the two uATGs.

14 uATGs were found in the 532 bp UTR of *GUS* mRNA from *Wip*_1737_*::GUS* transgenic tobacco lines. These uATGs form more uORFs than the six uATGs in 272 bp UTR of *GUS* mRNA from *Wip*_1231_*::GUS* transgenic lines. The fourth and fifth uATGs in the 272 bp UTR which can produce GUS fusion protein, are in frame with a termination code in the 532 bp UTR and form a uORF. Interestingly, the uORF also exists in the 361 bp UTR of *GUS* mRNA from *Wip*_1231a_*::GUS* transgenic tobacco lines if the lines have the same transcription start site as *Wip*_1231_*::GUS* lines. We suggest that the uORF may lead to the low GUS activity of transgenic plants transformed by pWip_1737_ and pWip_1231a_. We also suggest that the uORF might accelerate the degradation of mRNA from *Wip*_1731_*::GUS* and *Wip*_1500_*::GUS* transgenic tobacco lines, because the levels of mRNA in these lines were lower than that in *Wip*_1231_*::GUS* transgenic tobacco lines. We cannot exclude other factors that contribute to the attained results.

A few reports have shown that the CaMV 35S enhancer has the ability to alter the expression of nearby genes by affecting nearby promoters [14,30,31]. In the T-DNA region of transformation plasmids used for the promoter analysis, the selectable marker gene *hptII* was controlled with double enhanced CaMV 35S promoter. We confirmed that the 35S promoter influenced the *GUS* expression in *Wip*_1231_*::GUS* transgenic plants, because low GUS activity was observed when the 35S promoter was replaced with the NOS promoter. A problem may appear if the 35S promoter exists nearby an inducible or tissue-specific promoter that drives target transgene expression, but the problem might be negligible when the adjacent promoter is a strong constitutive one. The disadvantage of the 35S promoter can be overcome by using enhancer-blocking insulators to disturb the interaction between the 35S promoter and adjacent promoters [43]. Every coin has two sides; maybe the property of the 35S promoter can be used to develop a “strong constitutive” promoter controlling transgene expression in plants, such as the *Wip*_1231_ and several other truncated *Wip1* promoters studied in the paper, although these truncated *Wip1* promoters drive low transcriptional level of transgene.

## 4. Experimental Section

### 4.1. Isolation and Analysis of the Maize *Wip1* Promoter

Maize genomic DNA was isolated from leaves according to a previously described method [44]. A 1737 bp fragment designated as the full-length promoter sequence was amplified using the primers F1737 (5′-AACTGCAGGGCTCCGTTCTACTTGACT-3′) and R1737 (5′-CGGGATCCGGTCTCGGACGAGCTGTTCTT-3′). The underlined letters indicate *Pst*I and *Bam*HI restriction sites. Regulatory motifs were identified using PlantCARE [23] and PLACE [24], the transcription start sites were predicated on the website: http://www.fruitfly.org/seq_tools/promoter.html [25].

### 4.2. Plasmid Construction

The plasmid pBI221 was digested with *Hin*dIII and *Eco*RI. The CaMV 35S promoter-GUS-NOS fragment was purified and ligated into plasmid pCAMBIA1300 that had been digested with the same enzymes to construct the plasmid pCAMBIA1300-221. The amplified 1737 bp full-length promoter sequence was digested with restriction enzymes *Pst*I and *Bam*HI and inserted upstream of the *GUS* gene in plasmid pCAMBIA1300-221, replacing the CaMV 35S promoter to construct a new plasmid, pWip_1737_ (Figure S6). The full-length *Wip1* promoter was replaced with other truncated *Wip1* promoters to construct the other vectors. The details for the primers used to amplify the truncated promoters are shown in Figure 1 and Tables 1 and 2.

To construct vectors where 35S promoter was replaced with NOS promoter to control *hptII* selectable gene, NOS promoter was amplified using Gateway binary vector pGWB454 (AB294466.1) as template with primer F-NOS (5′-CCAACATGGTGGCATCATGAGCGGAGAATTAAG-3′) and primer R-NOS (5′-CTCGAGAGATCCGGTGCAGATTATTTG-3′). The underlined letters indicate *Bst*XI and *Xho*I restriction sites, respectively. The vector pWip_1231_ was digested with *Bst*XI and *Xho*I to produce three fragments, a 10 kb fragments containing vector bone, a 1.1 kb fragment containing *hptII* gene and a 0.8 kb fragment containing 35S promoter. The 10 and 1.1 kb fragments were gel purified separately and the 0.8 kb one was discarded. The 10 kb purified fragment ligated with the amplified NOS promoter fragment was digested by the same endonucleases to form the medium vector that was digested by *Xho*I. Then the purified product ligated with the 1.1 kb purified fragment in correct orientation to form the vector p1300-1231-NOS. The new control vector p1300-35S-NOS derived from pCAMBIA1300-221 was constructed similarly. The restriction endonuclease sites were in marked in detail in supplementary Figure S1.

### 4.3. Plant Transformation

For the tobacco plant transformations, the plant expression plasmids were transferred into competent *Agrobacterium tumefaciens* (strain LBA4404) cells by freeze-thaw treatment. The transformed *Agrobacterium* were selected on YEB-agar plates containing 100 mg/L kanamycin and 100 mg/L streptomycin. Recombinant *Agrobacterium* were infiltrated into the young tobacco (*Nicotiana tobacum*) leaves according to the described method [45]. For the *Arabidopsis* transformations, vector constructs were transformed into *Agrobacterium tumefaciens* strain GV3101. *Arabidopsis* ecotype Columbia Col-0 was transformed using the floral dip method [46]. The obtained tobacco and *Arabidopsis* T_0_ seeds were selected on MS medium plates containing 30 mg/L Hygromycin B to eliminate the non-transgenic plants.

For the rice transformation, vector constructs were transformed into *Agrobacterium tumefaciens* strain EHA105. Three-week-old calli derived from mature seed (*Rryza sativa* L. cv. Kita-ake) were used for the transformation. The transformation was performed according to the described method [47].

### 4.4. Wounding of Transgenic Plant Leaves

The second leaves from apex of 40-day-old T_3_ tobacco plants grown in greenhouse were wounded using three different kinds of methods. The first wound treatment was performed according to Walker-Simmons, *et al.* [26] wherein the leaves were crushed across the midvein by a hemostat, with a second wounding 20 h later, and the leave samples were harvested 24 h after the first wounding. The second wound treatment was performed according to An, *et al.* [27] wherein the leaves were cut into about 1 cm^2^ sections and the leaf slices were put in Murashige and Skoog (MS) liquid medium containing 3% sucrose (pH 5.8). The leaf slices were wounded by making several small holes in each section with forceps, and then were placed in tissue culture incubator at 28 °C for 24 h under light (3000 Lux), then the samples were harvested for the experiment. The third wound treatment was performed according to the method described by Xu, *et al.* [28] wherein the leaves were cut three times perpendicularly to the midvein by a scissor along both edges of the leaf blade without damaging the midvein. The first leaf under the wounding one was cut off before wounding as unwounded sample, frozen in liquid nitrogen and stored at −80 °C before extracting crude protein.

For the wounding of rice leaves, the second leaves from apex of 40-day-old T_2_ rice plants grown in greenhouse were wound according to the method described by Xu, *et al.* [28] wherein the leaves were cut at intervals of 1 cm perpendicularly to veins by a scissor along both edges of the leaf blade without damaging the midvein, and the leave samples were harvested 24 h after wounding. The first leaf under the wounding one was cut off before wounding as unwounded sample, frozen in liquid nitrogen and stored at −80 °C before extracting crude protein.

### 4.5. Analysis of RNA Level by qRT-PCR

The second leaf from the apex of 40-day-old T_3_ transgenic tobacco was sampled. Total RNA was extracted using *EasyPure*™ Plant RNA Kit (TransGen, Beijing, China). The RNA was reverse transcribed to cDNA using TransScript^®^ II First-Strand cDNA Synthesis SuperMix Kit (TransGen, Beijing, China), and qRT-PCR was performed using the TransStart^®^ Green qPCR SuperMix Kit from Beijing TransGen Biotech Co. Ltd. (Beijing, China). The primers used for qRT-PCR were designed according to the sequences of the *GUS* gene from vector pBI221 (AF502128.1) and tobacco *Actin* gene (U60495.1). The sense primer for *GUS* gene is S-GUS: 5′-CCAACTCCTACCGTACCTC-3′, and the antisense primer is A-GUS: 5′-TCGAAACCAATGCCTAAA-3′. The sense primer for *Actin* gene is S-ACTIN: 5′-AAGGGATGCGAGGATGGA-3′, and the antisense primer is A-ACTIN: 5′-CAAGGAAATCACCGCTTTGG-3′. The instrument of ABI 7300 was used. The reaction volume is 20 μL. The amplification program was: 95 °C 2 min for pre-denature, then 40 cycles of 95 °C 10 s for denature, 55 °C 30 s for annealing, 72 °C 31 s for elongation were followed. The fluorescence data was collected at the end of elongation and analyzed by the 2^−ΔΔCt^ method [29].

### 4.6. Analysis of the Transcription Start Sites by 5′ Rapid Amplification of cDNA Ends (5′-RACE)

According to the sequences of maize *Wip1* gene (X71396.1) and *GUS* gene from vector pBI221 (AF502128.1), two specific primers, wip1-R: 5′-CAAAAAGGACTGCGACCCCGTCTG-3′ and GUS-R: 5′-GTTCTGCGACGCTCACACCGATACC-3′, were designed. wip1-R was used to amplify the 5′ end of *Wip1* mRNA in maize. GUS-R was used to amplify the 5′ end of *GUS* mRNA in transgenic tobacco. Total RNA was extracted from leaves of 10-day-old maize inbred line Z31 and 30-day-old transgenic tobacco plants using EasyPure™ Plant RNA Kit (TransGen, Beijing, China). The generation of RACE-ready cDNA and rapid amplification of cDNA end were performed strictly according to the user manual of SMARTer™ RACE cDNA Amplification Kit (Clontech, Mountain View, CA, USA).

### 4.7. GUS Activity Assay and Histochemical Staining

T_2_ transgenic tobacco seeds were germinated on MS medium plates with 30 mg/L Hygromycin B to eliminate the non-transgenic plants. The surviving 14-day-old seedlings were immersed in GUS staining solution [48] and incubated overnight at 37 °C. Then, the samples were de-pigmented with 70% ethanol at 37 °C until the chlorophyll had completely disappeared.

For quantitative analysis, young leaves of 40-day-old T_3_ tobacco seedlings and 20-day-old T_3_*Arabidopsis* seedlings were homogenized in 500 μL extraction buffer (50 mM sodium phosphate buffer, pH 7.0, 10 mM EDTA, 0.1% Triton X-100, 10 mM β-mercaptoethanol) and clarified by centrifugation at 13,000 rpm for 10 min at 4 °C. 10 μL supernatant was used to assay the GUS activity in 100 μL extraction buffer containing 2 mM 4-methylumbelliferyl-β-D-glucuronide. Fluorescence was measured using Skanlt 2.4.3 RE for Varioskan Flash (Thermo, Waltham, MA, USA). Protein concentration was determined using the Model 680 spectrophotometer (Bio-Rad, Philadelphia, PA, USA) using the Coomassie Brilliant Blue method [49].

## 5. Conclusions

We used standard methods to study the function of the maize *Wip1* promoter and found that several truncated *Wip1* promoters led to high GUS activity in transgenic plants, even though the *GUS* transcriptional level was relatively low. The high GUS activity may be ascribed to high translation efficiency. The activity of the truncated *Wip1* promoter fragments were influenced by the adjacent 35S promoter, however they may also be used as strong constitutive promoters to drive transgenes in plant biotechnology when a 35S promoter exists nearby. This may be a way to develop new “strong constitutive” promoters.

## Supplementary Information



## Figures and Tables

**Table 1.** The sequence of the primers used in the paper.

Primers	Sequence
F1737	5′-AACTGCAGGGCTCCGTTCTACTTGACT-3′
R1737	5′-CGGGATCCGGTCTCGGACGAGCTGTTCTT-3′
F1231	5′-AACTGCAGTTTGTTTTGGATTATAAT-3′
F1191	5′-AACTGCAGCGCGTCGCCGTCTCTTACTGT-3′
F791	5′-AACTGCAGTTCTTTTTAGTTGTCGCTGGA-3′
F491	5′-AACTGCAGGGGAAGATTATTACTATACAC-3′
R1231	5′-CGGGATCCTTCCTCGTGATCATTTCCG-3′
F1231A	5′-AACTGCAGCCTTACATAGTGGTTGGT-3′
F1231B	5′-AACTGCAGGCTTCTATTTTATCACAC-3′
F1231C	5′-AACTGCAGGGCGTCCATTTGTATCTC-3′
R1231a	5′-CGGGATCCATGGATGGATAGACGCTT-3′
R1231b	5′-CGGGATCCGCCGAGATCTGAACATGC-3′
R1231c	5′-CGGGATCCCCTCTCGTAGAAGCAAGC-3′

**Table 2.** The primers used to amplify different truncated promoters.

The amplified fragment	Forward Primer	Reverse Primer
*Wip*_1500_	F1231	R1737
*Wip*_1231_	F1231	R1231
*Wip*_1191_	F1191	R1737
*Wip*_791_	F791	R1737
*Wip*_491_	F491	R1737
*Wip*_1231A_	F1231A	R1231
*Wip*_931_	F1191	R1231
*Wip*_1231C_	F1231C	R1231
*Wip*_1231a_	F1231	R1231a
*Wip*_1231b_	F1231	R1231b
*Wip*_1231c_	F1231	R1231c
*Wip*_1231Aa_	F1231A	R1231a
*Wip*_1231Ab_	F1231A	R1231b
*Wip*_1231Ac_	F1231A	R1231c
*Wip*_1231Ba_	F1231B	R1231a
*Wip*_1231Bb_	F1231B	R1231b
*Wip*_1231Bc_	F1231B	R1231c
*Wip*_1231Ca_	F1231C	R1231a
*Wip*_1231Cb_	F1231C	R1231b
*Wip*_1231Cc_	F1231C	R1231c
