# Supplementary material for: Characterization of a Maize Wip1 Promoter in Transgenic Plants
Source: Int J Mol Sci. 2013 Dec 6;14(12):23872–92. doi: 10.3390/ijms141223872 (PMC3876083; doi:10.3390/ijms141223872)
Supplement: Supplementary file 1 [file ijms-14-23872-s001.pdf]

Supplementary Information

Figure S1. Western blot analysis of GUS protein level in transgenic tobacco leaves. The antibody (ab50148) against GUS protein was purchased from Abcam Company (Shanghai, China). The second leaf from the apex of 40-day-old T_3 transgenic tobacco was collected as one sample from three independent transgenic lines. The samples were ground to powders in liquid nitrogen. The crude ptotein were extracted using sample buffer (S3401, Sigma, St. Louis, MO, USA). The supernatants were used for gel electrophoresis and Western blot. Lane **1**–**2**: Crude protein from $pWip_{1737}$ transgenic tobacco; Lane **3**: Crude protein from $pWip_{1500}$ transgenic tobacco; Lane **4**–**8**: Crude protein from $pWip_{1231}$ transgenic tobacco; Lane **9**–**13**: Crude protein from pCAMBIA1300-221 transgenic tobacco; Lane **14**: Crude protein from wide type tobacco; The target band was indicated by an arrow line.

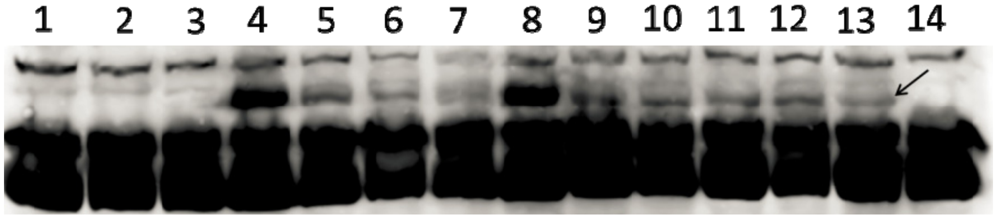

Figure S2. Analysis of enhancing translation motifs in 5'-UTR of *GUS* mRNA in $pWip_{1231}$ transgenic tobacco. Part of GUS coding sequence was indicated in bold italic letters, and part of 5'-UTR from pCAMBIA1300-221 vector born was indicated in italic letters. CAA trimers were indicated in bold letters, and the ACAAAA motif and motifs similar to ACAATTAC element were shown with underline.

CCTGATTAGATCGAGAGGGGAAGATTATTACTATACACTGTACATCACACTATATTCCATTGTT

TTACCTCATTGTGAATCGCACGCATGAAATGGTGAATAAGGCTAGGAT**CAA**T**CAA**CCGATGCT

TCTACCAGCTGCGCGT**CAA**GCCA**CAA**AACTGTGCTGATCGGATAACACGTCGTCGTCACTTC

CGTCCATTTTGGAC**CAA**ATACATACGCTGATGAGGATGATCGGAAATGATCACGAGGAA*GGA*

*TCCCCGGGTGGTCAGTCCCTT****ATGTTACGTCCTGTAGAAACCCCAACCCGTGAAATCAAAAA***

ACTC

Figure S3. uORF analysis of GUS mRNA in $pWip_{1231}$ transgenic tobacco. Part of GUS coding sequence was indicated in bold italic letters, and part of 5'-UTR from pCAMBIA1300-221 vector bone was indicated in italic letters. Every uORF was underlined, and the one form by the uATG in frame with initiating ATG of *GUS* gene was underlined by green arrow line.

CCTGATTAGATCGAGAGGGGAAGATTATTACTATACACTGTACATCACACTATATTCCATTGTT

TTACCTCATTGTGAATCGCACGCATGAAATGGTGAATAAGGCTAGGATCAATCAACCGATGCT

TCTACCAGCTGCGCGTCAAGCCACAAAACTGTGCTGATCGGATAACACGTCGTCGTCACTTC

CGTCCATTTTGGACCAAATACATACGCTGATGAGGATGATCGGAAATGATCACGAGGAA*GGA*

*TCCCCGGGTGGTCAGTCCCTT****ATGTTACGTCCTGTAGAAACCCCAACCCGTGAAATCAAAAA***

ACTC

Figure S4. uORF analysis of *GUS* mRNA in $pWip_{1737}$ transgenic tobacco. Part of GUS coding sequence was indicated in bold italic letters, and part of 5'-UTR from pCAMBIA1300-221 vector born was indicated in italic letters. Every uORF was underlined, and the uORF starting from the same uATG which is in frame with the initiating ATG of *GUS* gene in $pWip_{1231}$ transgenic tobacco, was underlined by red line.

CCTGATTAGATCGAGAGGGGAAGATTATTACTATACACTGTACATCACACTATATTCCATTGTTTTACCTC

ATTGTGAATCGCACGCATGAAATGGTGAATAAGGCTAGGATCAATCAACCGATGCTTCTACCAGCTGCG

CGTCAAGCCACAAAACTGTGCTGATCGGATAACACGTCGTCGTCACTTCCGTCCATTTTGGACCAAATA

CATACGCTGATGAGGATGATCGGAAATGATCACGAGGAAGAGATGGTTCGTTATGCCATACGACTCGC

CGCCATGAGTCAAAGTTCCGCCTAACAAAATGTATGAGCTGGAAGCGTCTATCCATCCATCGCCCCCA

AAGAAATGCCATTGCAGGAGGACTACACGGCGGCAGGCAATGCCATGCATGTTCAGATCTCGGCGCC

GCCTATAAGTAGCTTCCTCCAGTGCACTGCTGTTGGCGCAACGAAGCTTGCTTCTACGAGAGGATATAG

AGAGGAAGAACAGCTCGTCCGAGACC*GGATCCCCGGGTGGTCAGTCCCTT****ATGTTACGTCCTGTAGA***

AACCCCAACCCGTGAAATCAAAAAACTC

Figure S5. uORF analysis of *GUS* mRNA in pWip$_{1231a}$ transgenic tobacco. Part of GUS coding sequence was indicated in bold italic letters, and part of 5'-UTR from pCAMBIA1300-221 vector born was indicated in italic letters. Every uORF was underlined, the uORF starting from the same uATG which is in frame with the initiating ATG of *GUS* gene in pWip$_{1231}$ transgenic tobacco, was underlined by red line. The uORF underlined by green arrow line could produce fusion protein with GUS.

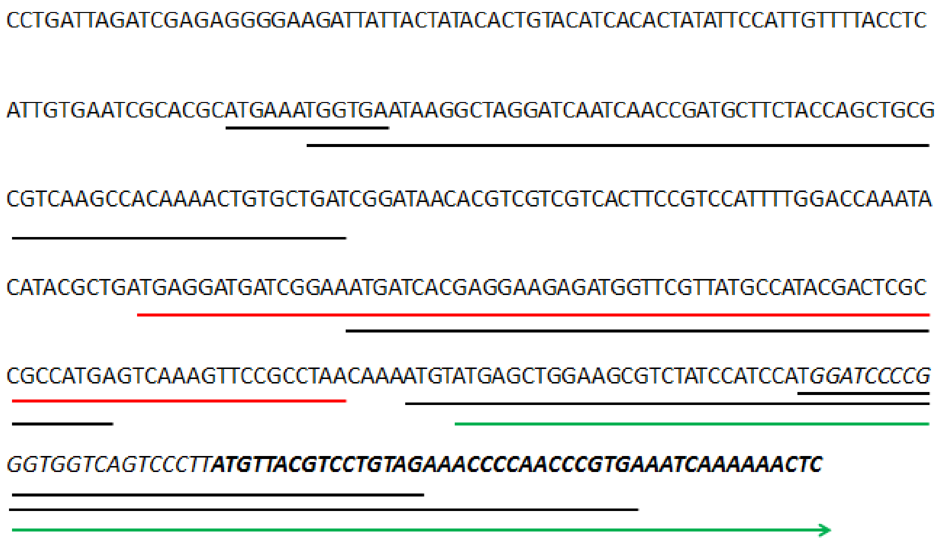

Figure S6. The diagram of T-DNA region in expression vector pWip$_{1737}$.

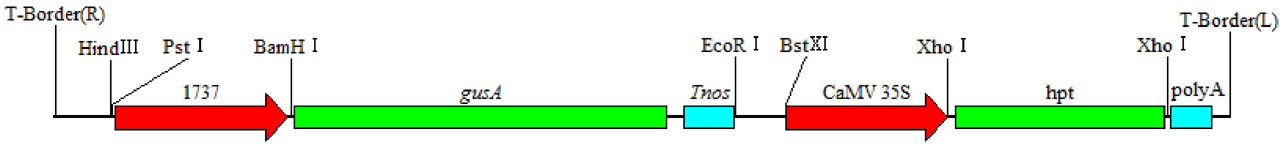

© 2013 by the authors; licensee MDPI, Basel, Switzerland. This article is an open access article distributed under the terms and conditions of the Creative Commons Attribution license (http://creativecommons.org/licenses/by/3.0/).
